# Changes in gut microbiota and metabolism associated with phenotypic plasticity in the honey bee *Apis mellifera*

**DOI:** 10.3389/fmicb.2022.1059001

**Published:** 2022-12-09

**Authors:** Duan C. Copeland, Patrick W. Maes, Brendon M. Mott, Kirk E. Anderson

**Affiliations:** ^1^USDA-ARS Carl Hayden Bee Research Center, Tucson, AZ, United States; ^2^Department of Microbiology, School of Animal and Comparative Biomedical Sciences, University of Arizona, Tucson, AZ, United States; ^3^Department of Entomology and Center for Insect Science, University of Arizona, Tucson, AZ, United States

**Keywords:** phenotypic plasticity, gut microbiota, precocious foragers, immunity, oxidative stress, vitellogenin, age-polyethism

## Abstract

Honey bees exhibit an elaborate social structure based in part on an age-related division of labor. Young workers perform tasks inside the hive, while older workers forage outside the hive, tasks associated with distinct diets and metabolism. Critical to colony fitness, the work force can respond rapidly to changes in the environment or colony demography and assume emergency tasks, resulting in young foragers or old nurses. We hypothesized that both task and age affect the gut microbiota consistent with changes to host diet and physiology. We performed two experiments inducing precocious foragers and reverted nurses, then quantified tissue-specific gut microbiota and host metabolic state associated with nutrition, immunity and oxidative stress. In the precocious forager experiment, both age and ontogeny explained differences in midgut and ileum microbiota, but host gene expression was best explained by an interaction of these factors. Precocious foragers were nutritionally deficient, and incurred higher levels of oxidative damage relative to age-matched nurses. In the oldest workers, reverted nurses, the oxidative damage associated with age and past foraging was compensated by high Vitellogenin expression, which exceeded that of young nurses. Host-microbial interactions were evident throughout the dataset, highlighted by an age-based increase of *Gilliamella* abundance and diversity concurrent with increased carbonyl accumulation and CuZnSOD expression. The results in general contribute to an understanding of ecological succession of the worker gut microbiota, defining the species-level transition from nurse to forager.

## Introduction

The ecological success of eusocial insects is attributed to an organized and efficient division of labor (Oster and Wilson, 1978). Social insects solve complex problems with individual behaviors, resulting in emergent group properties (Hölldobler and Wilson, 2008). The numbers of workers performing a particular task is optimized by feedback loops to efficiently collect, process, and distribute resources among colony members (Fewell, 2003). During normal ontogeny, individual workers transition among various tasks during their lifetime, and exhibit a broad range of phenotypic plasticity. More simply, colony demography is socially regulated (Huang and Robinson, 1996), allowing a proximate internal response to unpredictable external environments. Various worker tasks involve different physiological and behavioral demands, producing strong selection on social phenotypes. Social insects are well suited to the study of sociality and phenotypic plasticity because they represent a complex adaptive system or “superorganism” from which the functional parts can be manipulated and measured (Hölldobler and Wilson, 2008).

Honey bees are highly social insects that live in complex societies consisting of one reproductive queen and thousands of facultatively sterile workers. While the queen spends a preponderance of her life laying eggs, workers build and maintain all aspects of the hive. Under normal conditions, worker bees display age polyethism, performing tasks within the hive for the first 2–3 weeks of adult life before transitioning to outside tasks (Seeley, 1982). Specifically, young adults function as “nurse bees” that feed growing larvae, then act as food processors in middle age. Near 20 days of age, middle-age bees transition into foragers that then procure nectar (carbohydrates), pollen (protein and lipids), propolis (antimicrobial plant resins), and water (Seeley, 1982) from the pollination environment. Despite this well-established pattern, adult workers can decouple age from behavioral task in response to social cues from other workers and temporal effects in the pollination environment (Huang and Robinson, 1992; Johnson, 2010). Thus, the ontogeny of an adult bee is extremely plastic and nursing/foraging behaviors can be accelerated, slowed, or reversed (Robinson, 1992).

Phenotypic plasticity in honey bees workers is directly associated with the availability of nutrition and storage proteins, vitellogenin in particular (Amdam et al., 2003). Vitellogenin (Vg) is a phospholipoglyco-protein evolved to serve many functions; as an antimicrobial, antioxidant, and to produce brood food in the nurse worker head (hypopharyngeal) glands (Seehuus et al., 2007; Amdam, 2011). Associated with changes in the gut microbiota, foragers switch to a diet of simple sugars to support the metabolic demands associated with foraging (Anderson et al., 2018). This labor transition is associated with reduced lipid stores (Toth and Robinson, 2005), reduced Vg titers (Fluri et al., 1982), decreased nutritional status (Ament et al., 2008), differential gene expression (Byhrø et al., 2019), and protein oxidation; a direct measure of biological aging (Fedorova et al., 2014). Many differences contribute to foraging success; a decrease in body mass and a proportional increase in flight capacity (Vance et al., 2009). However, orientation to the pollination environment is the riskiest time of an adult bee’s life. A recent study documented that 40% of bees die during the pre-foraging stage of life, a time where bees perform exploratory and learning orientation flights (Prado et al., 2020). Bees that survive this training face a constant increase in extrinsic mortality risk per unit time that increases to 100% after 18 days of foraging activity (Dukas, 2008), yet only ~20% of foragers will live past 10 days of foraging (Visscher and Dukas, 1997). Therefore, the age a worker initiates foraging has a strong impact on an individual’s lifespan and colony fitness.

Foraging also has direct consequences for intrinsic senescence, including increased sensitivity to physiological stressors (Remolina et al., 2007) and a decrease in innate immune defenses (Amdam et al., 2004, 2005; Schmid et al., 2008; Lourenço et al., 2019). Foragers also show an increased susceptibility to oxidative stress (Seehuus et al., 2006), including oxidative damage to the brain (Rueppell et al., 2007), trophocytes, and fat cells (Hsieh and Hsu, 2011). The accumulation of oxidative damage from reactive oxygen species (ROS) is proposed as the main cause of aging (Harman, 1956). Thus, a precocious transition to foraging is predicted to result in premature aging. Flight and the associated ROS accumulation from muscle usage and attrition may surpass the capacity for antioxidant enzymes to remove them. Indeed, the honey bee’s innate antioxidant enzymes: e.g., various superoxide dismutases, catalase, and glutathione S-transferase, reach their greatest expression in older workers (Corona et al., 2005). While the physiology of behavioral plasticity and aging has been explored in honey bees, the role of the gut microbiome in this process is poorly known (Vonaesch et al., 2018). A compendium of results characterizing the transition to foraging found that the worker hindgut microbiota is depleted of core hindgut *Lactobacillus* firm4, firm5, and *Bifidobacterium asteroides,* and can be enriched for Acetobacteraceae Alpha 2.1 and *Bartonella apis* (Anderson et al., 2018), but results were inconclusive for core ileum species perhaps reflecting a lack of tissue-specific sequencing.

The honey bee gut microbiota is remarkably consistent and dominated by five omnipresent, highly co-evolved phylotypes representing >95% of bacterial cells; *Lactobacillus*, Alphaproteobacteria, Betaproteobacteria, Gammaproteobacteria, and *Bifidobacterium* (Martinson et al., 2012; Sabree et al., 2012; Kwong and Moran, 2016). Recent work has revealed a strong association of the microbiome with worker physiology including the expression of insulin-like peptides and vitellogenin (Engel et al., 2016; Maes et al., 2016; Kešnerová et al., 2017; Raymann et al., 2017; Ricigliano et al., 2017; Zheng et al., 2017; Powell et al., 2021). Although the microbiota of nurses and foragers is taxonomically similar (Corby-Harris et al., 2014), composition differs by behavioral task and may impact host physiology and health (Anderson et al., 2018; Jones et al., 2018).

Here we investigate changes in the gut microbiota and host gene expression associated with typical and atypical ontogeny. Individual worker behavior and physiology can be manipulated *via* the perturbation of social structure (Huang and Robinson, 1996). We manipulated colony social structure to test two hypotheses: (1) Premature foraging comes with a physiological costs reflected in the gut microbiota, and (2) a return to nursing behavior in old age restores youthful physiology and associated microbiome characteristics. To test if gut microbiota differences are associated with ontogeny (atypical vs. typical) or age, we (1) generated “single-cohort colonies” (SCC) comprised of bees that were all the same age (Robinson et al., 1989) and (2) generated observation hives where we induced foragers to return to nursing behaviors. These perturbations of colony demography induce portions of the population to assume behavioral tasks independent of age. For the first experiment, we assessed differences in nurses and precocious foragers (PF) midgut and ileum gut microbiota of the same age, monitoring fat body gene expression related to immunity and oxidative stress. Likewise, we assessed protein oxidation in the fat body resulting from precocious foraging. In the second experiment, we assessed the hindgut (ileum and rectum) microbiota and fat body gene expression of reverted nurses relative to normal worker ontogeny. PF midgut and ileum microbiotas were explained by both age and ontogeny, but gene expression of the fat body tissue was best explained by an interaction of ontogeny and age. Precocious foragers lacked key ileum species *Gilliamella*, and accrued more oxidative damage relative to age-matched nurses and foragers that experienced normal ontogeny. The hindgut microbiotas of reverted nurses were remarkably stable, but relative to young nurses, vitellogenin expression was significantly elevated, and carbonyl accumulation increased by an order of magnitude.

## Materials and methods

### Colony manipulations and sampling

**Figure 1:**
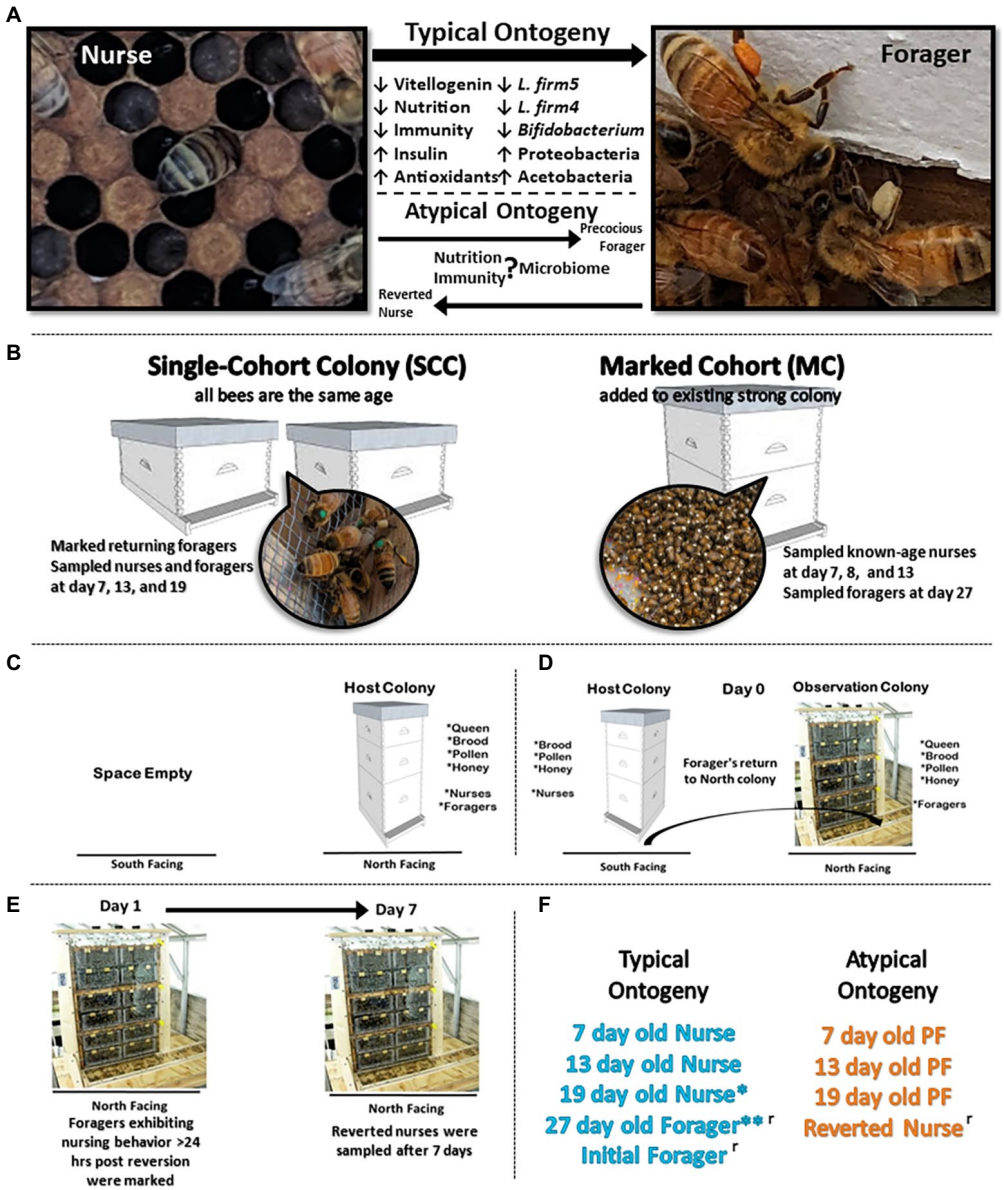
Experimental design. **(A)** During typical ontogeny workers transition from in-hive tasks like nursing to riskier outside the hive tasks like foraging as they age. We designed two experiments to explore this relationship of ontogeny, phenotypic plasticity, physiology, and the gut microbiota. **(B)** Experimental design for precocious forager experiment: to decouple age and behavioral task, we created two single-cohort colonies (SCC) with 4,500 and 3,500 bees. In a SCC, all bees are the same age but as an emergent property of sociality, some will differentiate by task into precocious foragers. We also established a marked cohort to sample normal age bees following classic ontogeny patterns. **(C–E)** Experimental design for reverted nurse experiment: a host colony was switched to an observation colony so that returning foragers returned to a colony with no nurses. By the same social mechanism, a proportion of old foragers will revert to nursing behaviors. **(F)** Typical (age-right and blue color) ontogeny occurs under normal colony demography. Atypical (orange color) designates a decoupling of age and behavior in response to colony needs. *19 day old nurses can be considered as overage nurses, especially in a busy pollination season. These data present 19 are typical considering a long transition in-hive nursing before transitioning to foraging. **27 day old foragers were used in both experiments. ʳ designates categories used in reversion experiment.

To investigate changes in host physiology and gut microbial composition associated with the range of worker phenotypic plasticity, we designed and implemented two experiments in June 2019 at the USDA-ARS Carl Hayden Bee Research Center in Tucson Arizona (Figure 1A). The first experiment utilized a “single-cohort colony” (SCC) design, which was previously shown to uncouple behavioral task from chronological age (Robinson et al., 1989). We induced a subset of the population to become precocious (young) foragers (PFs). In the second experiment we removed the young (nurse) bee population, forcing older foragers to revert back to nursing behaviors.

Experiment 1 (Precocious foragers PF): Closed brood frames were sourced from 30 honey bee (*Apis mellifera, linguistica*) hives. Frames were incubated overnight (30°C, 75% relative humidity, and 24 h dark cycle) and newly emerged adult workers were collected and combined into a mixed cohort to implement into experiment. To construct the two SCCs, newly emerged workers (4,500 and 3,500) were added to a small hive box, each containing a naturally mated queen, one frame with pollen and honey, and one frame with eggs and open brood (Figure 1B). Bees assigned to each SCC were <24 h old and differentiated into separate behavioral tasks, i.e., nurses and atypically, PFs. Additionally, a marked cohort (MC) with 4,100 newly emerged workers was constructed to serve as a control for sampling normal ontogeny throughout both experiments (Figure 1B). Newly emerged workers were marked with paint on their thorax and transferred into a healthy double-deep colony free from visible signs of disease. This allowed us to sample natural age nurses (7D, 8D, and 13D) and 27 day (27D) old natural foragers, complementing both experiments. On day 6 of the experiment, SCC PFs we observed with corbicular pollen loads were marked with paint on their thorax.

At the peak of foraging activity in the summer, a workers lifespan is ~30 days (Fluri et al., 1982). Over the course of the experiment, we sampled marked foragers and nurses from the SCCs at 7, 13, and 19-days old. By design, SCC nurses and foragers performed the same behavioral tasks for the duration of the experiment. Nurses were identified by observing the brood nest and sampling bees that spent 3 s with their head in a cell containing brood. Part way through the experiment, we replaced the brood frame in the SCC’s to ensure no newly emerged workers replaced the current nurses. For the MC sampling, we sampled age-right nurses at 7, 8, and 13-days old and age-right foragers at 27-days based on well-established honey bee worker ontogeny (Seeley, 1982). Thus all samples could be categorized as typical (age-right) or atypical (PFs) ontogeny (Figure 1F). Sampled bees were collected with sterile soft forceps, snap frozen with dry ice, and stored in −80°C for processing.

Experiment 2 (Reversion REV): Three putatively healthy double-deep colonies were used to induce foragers to revert to nurse behaviors (Figure 1C). A mobile shed was retrofitted with 6 stalls, 3 north facing and 3 south facing to accommodate the colonies. Each stall was provided a single entrance by drilling a hole through the sheds wall. A 0.5 m flexible plastic tube was inserted through the hole and attached to the colony’s bottom board. Colonies were installed in the north facing stalls and given 1 week to acclimate to the new location. The night prior to the beginning of the experiment, each colony was moved to south facing stalls (Figure 1D). The vacant north facing stalls were replaced with three-frame observation colonies containing the queen from the source colony, one frame of food (stored honey and pollen), one frame of uncapped brood (to ensure no emergence would occur), and an empty frame to provide room for the queen to lay. A one-way entrance reducer was installed on the south facing source colony. The next morning, foragers leaving the source colonies returned to the observation colonies at the northern entrance.

We sampled groups of initial foragers (IF), defined by having corbicular pollen loads on their legs that returned to the north facing observation hives. The observation colonies composed of a queen and foragers were given 24 h to adjust to the new colony demography and redistribute into nurse and foragers. Next, individuals observed engaged in nursing behavior (head in cell with larvae >3 s) were painted on the thorax. After 1 week, previously marked individuals that were still observed engaged in nursing behaviors were sampled as reverted nurses (RN; Figure 1E).

Worker dissections occurred under sterile conditions. The sting was discarded and the fore and hindguts were removed from the abdomen. Gut tissues were dissected in 95% ethanol to wash and separate the midgut, ileum, and rectum before being added to a bead-beating tubes with 0.2 g of 0.1-mm silica beads and 600 μl of 1X TE buffer. Experiment 1 focused on the midgut and ileum tissues. Experiment 2 focused on the ileum and rectum. Both experiments utilized the abdominal fat body and attached dorsal sclerites as a single unit for gene expression and protein oxidation (carbonyl) assay to quantify biological aging.

### DNA/RNA extractions

In preparation for DNA/RNA extractions, samples were bead-beaten for 2 min at 30-s intervals and centrifuged to recover the supernatant. Gut tissue DNA was extracted with Thermo Scientific GeneJET Genomic DNA Purification Kit (Thermo Fisher Scientific, Waltham, Massachusetts, United States). Fat body supernatant was split into two aliquots of 300 μl, one used for RNA extraction [Thermo Scientific™ GeneJET Genomic RNA Purification Kit (Thermo Fisher Scientific, Waltham, Massachusetts, United States)] and the other reserved for carbonyl assay. The extracted fat body RNA was converted into cDNA with Thermo Scientific RevertAid First Strand cDNA Synthesis Kit (Thermo Fisher Scientific, Waltham, Massachusetts, United States). DNA fractions for each sample were used for 16S rDNA amplicon sequencing and bacterial quantification *via* real-time quantitative-PCR (qPCR). RNA fractions were used to create cDNA and examine gene expression *via* qPCR.

We quantified total bacterial abundance for gut tissues with a qPCR assay of bacterial 16S and fungal 18S rRNA gene copies (Liu et al., 2012a,b). The bacterial 16S gene template was amplified using forward primer 27F (5′-AGAGTTTGATCCCTCAG-3′) and reverse primer 1522R (5′-AAGGAGGTGATCCAGCCGCA-3′). The fungal 18S gene template was amplified using forward primer PanFungal_18S_F (5′-GGRAAACTCACCAGGTCCAG-3′) and reverse primer PanFungal_18S_R (5′-GSWCTATCCCCA KCACGA-3′). Quantitative PCRs for 16S rRNA genes were carried out in triplicate on a BioRad CFX96 thermocycler in 12 μl reactions containing 5 μl of New England Biolabs – Luna^®^ Universal Probe qPCR Master Mix (New England Biolabs, Ipswich, Massachusetts, United States), 0.5 μl forward primer, 0.5 μl reverse primer, 4 μl of H_2_O and 2 μl of DNA template. The cycling conditions were 95°C for 3 min followed by 40 cycles of 95°C for 10 s and 60°C for 60 s. The qPCR results were expressed as the total number of 16S and 18S rRNA gene copies per DNA extraction (100 μl volume elution).

To provide absolute quantification of 16S and 18S rRNA copy number and ensure inter-run comparability, in-run standard curves and no-template controls were included on each run. Invitrogen’s pCR™2.1 TOPO™ (Thermo Fisher Scientific, Waltham, Massachusetts, United States) was used to produce plasmid vectors, which were then transformed into DH5α™ cells (Thermo Fisher Scientific, Waltham, Massachusetts, United States). Successfully transformed colonies were selected and grown overnight in broth. Plasmid DNA was purified using the Thermo Scientific GeneJET Plasmid Miniprep Kit (Thermo Fisher Scientific, Waltham, Massachusetts, United States). The purified plasmid cells were measured using an Implen nanophotometer P300, and the known mass of plasmid plus PCR insert was used to calculate 16S plasmid-standard copies per μl. A 10-fold serial dilution of the plasmid standards was included on each plate, and these data were pooled across all plates to calculate a single standard curve used to interpolate all sample Cq values. To determine the total number of 16S rRNA-gene copies present in each sample extraction, Cq values were adjusted for elution volume and any subsequent dilution(s).

### 16S rRNA gene amplicon sequencing and analysis

A 466-bp fragment in the V3–V4 region of the 16S rRNA gene was amplified using PCR primers (forward primer, 341F 5′- CCTACGGGNGGCWGCAG-3′; reverse primer, 805R 5′-GACT ACHVGGGTATCTAATCC-3′). DNA library preparation was performed following Illumina MiSeq DNA library preparation protocol. Sequencing was performed at the University of Arizona Genetics Core (UAGC) on a MiSeq following the manufacturer’s guidelines. The sequence data for this study have been deposited in GenBank, Sequence Read Archive no. as (PF) PRJNA801240 and (Reversion) PRJNA885470.

The 16S rRNA gene sequences were processed by gut tissue using MOTHUR v.1.44.3 (Schloss et al., 2009). Briefly, forward and reverse reads were joined using the make.contigs command. After the reads were joined, the first and last five nucleotides were removed using the SED command in UNIX. Sequences were screened to remove ambiguous bases, using the screen.seqs command. Unique sequences were generated using the unique.seqs command. A count file containing group information was generated using the count.seqs command. Sequences were aligned to BEExact (Daisley and Reid, 2021) database using the align.seqs command. Sequences were filtered to remove overhands at both ends and gaps using filter.seqs. The unique.seqs command was ran again to remove new redundancies from filtering. A precluster step using pre.cluster was performed. Chimeras were removed using chimera.uchime command (Edgar et al., 2011). Sequences were classified with the BEExact database using classify.seqs command. Sequences that were not bacterial origin were removed using the remove.seqs command. All unique sequences with one or two members (single/doubletons) were removed using the AWK command in UNIX. A distance matrix was constructed for the aligned sequences using the dist.seqs command. Sequences were classified at the unique level with the BEExact database. Uniques were merged at the species-level with the merge.otus command. Samples with <5,000 reads were excluded from downstream analyses. ASVs at the species-level that were left unclassified by BEExact but matched unambiguously at 100% identity to genus were assigned as “*genus* unclassified.”

### Gene expression

The fat body is a main metabolic tissue of the honey bee and is functionally analogous to vertebrate liver or adipose tissue (Liu et al., 2009). Comparisons of fat body gene expression can relay information on immunocompetence and overall health. A list of genes used in both experiments can be found in Supplementary Table S1. Quantitative PCR reactions for immune gene expression were performed in triplicate as follows: initial denaturation at 95°C for 5 min; 40 cycles with denaturation at 95°C for 15 s; and a primer-pair-specific annealing and extension temperature for 30 s. To confirm the absence of contaminating genomic DNA and primer dimers in the qPCR assay, we monitored amplification and melting curves. Relative gene expression was determined based on standardized Ct values (Δ Ct; Livak and Schmittgen, 2001) using the mean of two reference genes: *β-actin* and *RPS18*. *β-actin* and *RPS18* are constitutively expressed in different honey bee tissues and has been previously established as an effective control for calibrating less constitutive gene expression in adult workers (Evans et al., 2013; Jeon et al., 2020).

### Carbonyl assay

To measure protein damaged by oxidative stress, we quantified the accumulation of protein carbonyl groups *via* another well-validated assay (Reznick and Packer, 1994). To determine carbonyl content of fat body homogenates, we used Protein Carbonyl Content Assay Kit (MAK094; Sigma-Aldrich, Burlington, Massachusetts, United States). Briefly, samples were treated with a 10 mg/ml streptozocin solution and incubated for 15 min to precipitate nucleic acids. Keeping the supernatant, 2,4-dinitrophenylhydrazones (DNPH) was added to samples to form stable dinitrophnyl hydrozone adducts. Derivatized proteins were precipitated with trichloroacetic acid and were followed by three successive ice-cold acetone washes. Samples were resuspended in 100 μl of 6 M guanidine (pH 2.3). The total protein concentration of each sample was measured using a Pierce™ BCA Protein Assay Kit (Thermo Fisher Scientific, Waltham, Massachusetts, United States; Smith et al., 1985). Protein oxidation was expressed as nanomoles of carbonyl groups per mg of protein.

### Statistical analysis

We evaluated both relative and absolute abundance to emphasize different properties of the microbiome data (Anderson et al., 2018). ASVs were normalized by 16S rRNA gene copy number *via* ribosomal RNA operons (*rrn*) database (Stoddard et al., 2015) and total bacterial 16S rRNA gene copies from qPCR prior to analysis. In this case, qPCR-normalized abundance is extrapolated from relative abundance of amplicons, so remains compositional, so may be best referred to as normalized abundance. To allow the use of parametric multivariate analyses (Pearson, 1897), we converted the qPCR-normalized bacterial abundances to ratios among all ASVs (Gloor and Reid, 2016) using the software CoDaPack’s centered log ratio (CLR) transformation (Comas and Thió-Henestrosa, 2011). After conversion, nearly all bacterial species followed normal distributions. Thus, a MANOVA on CLR transformed data considers changes of ASV abundance relative to the entire community, and the Wilcoxon results analyze absolute abundance of OTUs without reference to other community members (Anderson et al., 2018).

For the PF experiment a two-way MANOVA was performed on the CLR-adjusted abundances and Log10 normalized gene expression which allows for comparisons between dependent (ASVs or genes) and independent (age, ontogeny, and age*ontogeny) variables. For REV experiment we evaluated relative microbiota structure using a one-way MANOVA. We applied Pillai’s Trace test statistic; robust to violations of multivariate normality and homogeneity of covariance, followed by a False Discovery Rate (FDR) to account for multiple comparisons. We also performed principle component analysis (PCA) on the matrix of CLR scores for each gut tissue, to visualize the relationship of bacterial community composition with behavioral task and age-associated succession. To determine differences in absolute abundance of the microbial communities, we used Wilcoxon rank sum tests corrected for multiple comparison with FDR. Absolute abundance was used to determine correlations between bacteria using Spearman’s *ρ*, corrected by FDR for multiple comparisons.

We evaluated bacterial and fungal copy numbers by ontogeny (atypical versus typical) using one-way ANOVA with Tukey’s HSD post-hoc. Gene expression was log10 transformed to normalize variation and analyzed by sample type using one-way ANOVA with Tukey’s HSD post-hoc. PCAs of normalized gene expression was used to plot the relationship of immunity and oxidative stress genes for each experiment. For the REV experiment, a canonical correlation analysis was performed on log10 transformed gene expression. We compared carbonyl content by sample type using one-way ANOVA with Tukey’s HSD post-hoc. To validate the marked cohort (MC) as a control for typical ontogeny, we compared SCC age-right nurse microbiota and gene expression with the MC using Wilcoxon 2-sample *t*-test with FDR corrections (Supplementary Table S2). Multivariate analyses were conducted on ASVs with gene expression and carbonyl contents using Spearman’s *ρ* to find significant correlations after correcting for multiple comparisons with an FDR. All analyses were conducted in JMP_v14.3.0 (JMP_1989–2007) and/or SAS_v9.4 (Institute, SAS, 2015). We considered values of *p* < 0.05 statistically significant.

## Results

### 16S rRNA gene sequencing

Next-generation sequencing returned 23.9 million quality trimmed reads (455 bp assembled) across 469 libraries. Libraries used in downstream analyses were sampled to exhaustion according to goods coverage (>0.99%). The worker midguts (PF experiment) represented 5.0 million reads averaging 36.6 K per library. The worker ileums (used in both experiments) represented 13.2 million read averaging 55.9 K per library. The worker rectums (REV experiment) represented 5.6 million reads averaging 58.8 K per library. To examine the effect of community size in the midgut, the top 13 ASVs and a sum of remaining ASVs were used for downstream analysis. It should be noted that ASV 1–13 accounted for 84.5% of all sequences, the 14th group consisted of “SumOther” (Σ ASVs 14–740) accounted for the remaining 15.5%. In the ileum and rectum, the top 15 ASVs and a sum of remaining ASVs were also calculated for downstream analysis. In the ileum, ASV 1–15 accounted for 94.7% of sequences, while the 16th group of “SumOther” (Σ ASVs 16–214) accounted for the remaining 5.3%. ASV 1–15 in the rectum accounted for 88.9% of sequences, and the 16th group of “SumOther” (Σ ASVs 16–123) accounted for the remaining 11.1%.

**Figure 2:**
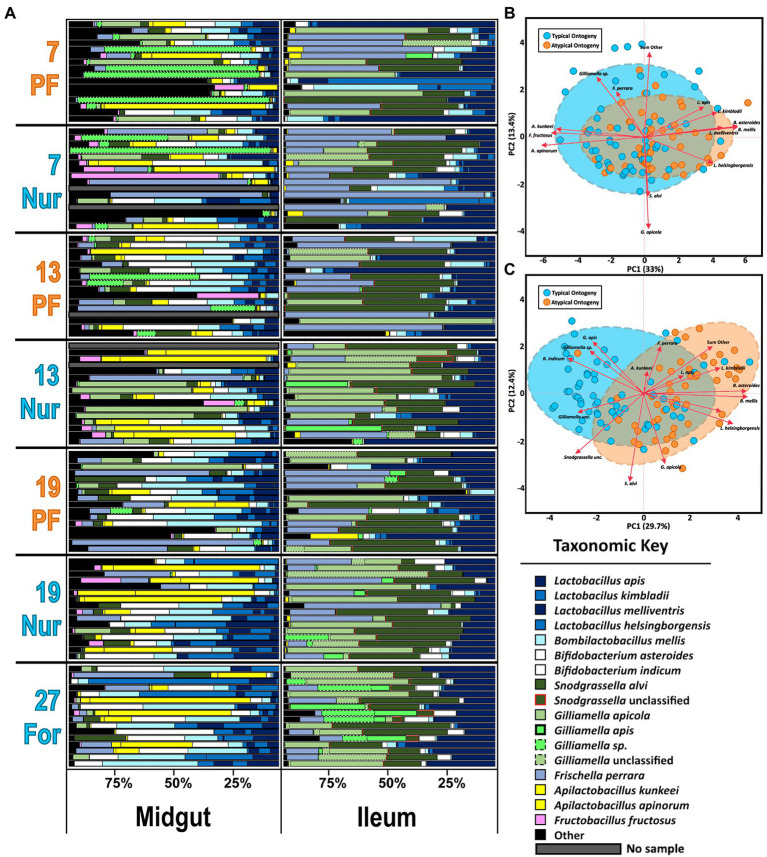
**(A)** The honey bee microbiota of the midgut and ileum by age and task. Color-coded bars represent relative abundance corrected by species-specific 16S rRNA gene copy number. Black represents diversity abundance, midgut: ASV’s 14–740 and ileum: ASV’s 16–214. **(B,C)** Principal component analysis of the midgut and Ileum, respectively, based on the most abundant ASVs. Clustered groups of points contain similar groupings of taxa with similar microbiota ratio abundances. Longer ASV vectors result from greater variation in CLR-adjusted scores. Density ellipses cover 90% of plots for each group.

Based on classification with BEExact, the PF midguts (Figure 2A; Supplementary Figure S1) the *Lactobacillus* Firm5 species cluster separated into 4 species: *L. apis*, *L. kimbladii*, *L. melliventris*, and *L. helsingborgensis.* Also common in the midgut, *Gilliamella* and *Apilactobacillus* separated into two species, and *G. apicola*, and *Gilliamella* sp., *A. kunkeei*, and *A. apinorum, respectively. Bifidobacterium asteroides*, *Bombilactobacillus mellis* (*L.* firm4), *Snodgrassella alvi*, *Frischella perrara*, and *Fructobacillus fructosus* were each represented by single species, however *F. fructosus* is the only species traditionally not considered core to the microbiome (Martinson et al., 2012; Sabree et al., 2012; Kwong and Moran, 2016). PF and REV ileum microbiotas (Figure 2A; Supplementary Figures S1, S2) were clustered together which resolved the same 4 *Lactobacillus* species and four distinct groups of *Gilliamella*: *G. apicola*, *G. apis*, *Gilliamella* sp., and *Gilliamella* unclassified. *Bifidobacterium* and *Snodgrassella* clustered into 2 species each: *B. asteroides*, *Bifidobacterium indicum*, *S. alvi*, and *S.* unclassified respectfully. The remaining species were represented by a single species each: *B. mellis*, *F. perrara*, and *A. kunkeei*. REV rectums (Supplementary Figure S3) were represented by the same four *Gilliamella* and *Lactobacillus* in the ileum with the exception of a 5th *Lactobacillus* that was included as *Lactobacillus* unclassified. *Bombilactobacillus* was represented by both traditionally known Firm4 species: *B. mellis* and *B. mellifer*. *Bifidobacterium asteroides*, *S. alvi*, *F. perrara*, and *Bartonella apis* were each represented by a single species.

For the PF experiment, the MC and SCC age-right nurses gut microbiota were compared and did not differ statistically at 7 and 13 days of age in the midgut and ileum (Supplementary Table S2). Additionally, a comparison between both SCC1 and SCC2 colonies and ASVs found some statistically significant differences in the midgut, but none for the ileum (Supplementary Table S3). Specifically, PFs from SCC1 had higher absolute abundances of *A. kunkeei*, *F. perrara*, *F. fructosus*, and *A. apinorum* at 7 days old and *F. fructosus*, *A. apinorum*, and “SumOther” at 13 days old. At 19 days, nurses in SCC1 had less *A. kunkeei*, *F. fructosus*, and *A. apinorum* than SCC2.

### Microbiota and gene associations with age and ontogeny

The two-way MANOVA performed on PF experiment midguts and ileums revealed significant variation due to age and ontogeny, but not as an interaction factor (Supplementary Table S4). In the midgut, the MANOVA revealed significance for six of the bacterial species analyzed. The independent variables (IV) of age (*F* value 3.23, Pr > *F* = 0.0001) and ontogeny (*F* value 2.57, Pr > *F* = 0.0047) were significant for six and five species, respectively. Specifically, *Gilliamella* sp. was abundant with age only and highest in 27D age-right foragers and 19-day nurses (Supplementary Figure S6) which also had the largest proportions and the largest microbiotas based on 16S rRNA gene copies (Supplementary Figure S4A). The fungal loads were more similar between sample types and tissues but bifurcated by age and task in the midguts; the oldest nurses and 27D foragers had the greatest fungal loads (Supplementary Figure S4B). *Apilactobacillus kunkeei* was explained by ontogeny and in greater abundances than atypical PF. *Fructobacillus fructosus* was strongly correlated with *A. kunkeei* (Supplementary Table S5: Spearman’s *ρ*, *r_s_* = 0.81, *p* = 0.0002), but was explained by both age and ontogeny; seemingly increasing with age and in greater proportions during typical ontogeny. *Bifidobacterium asteroides*, *B. mellis*, and *L. melliventris* were highly correlated across all samples (Supplementary Table S5: Spearman’s *ρ*, *r_s_* < 0.77, *p* = 0.0002), but had higher relative abundance in PFs versus age-matched nurses as well as increasing with age. PCA on CLR adjusted midgut microbiota groups well by ontogeny, with 33% of the variation captured by the first component and 13.4% by the second component (Figure 2B). Grouping is also consistent on microbial based age-association (Supplementary Figure S5). Patterns predicted by MANOVA (Supplementary Table S4) are represented in the midgut PCA (Figure 2B) with *Apilactobacillus* and *F. fructosus* being more associated with typical ontogeny and the highly correlated species, *Lactobacillus*, *Bifidobacterium*, and *Bombilactobacillus* were more associated with the guts of atypical ontogeny bees.

A visual inspection of the ileum’s relative abundances shows remarkable stability across age and ontogeny compared to the midgut (Figure 2A). The size of the ileum microbiota was mostly consistent across treatment groups (Supplementary Figure S4C). Nevertheless, the two-way MANOVA revealed significant variation by age (*F* value 2.48, Pr > *F* = 0.0001) and ontogeny (*F* value 4.47, Pr > *F* = 0.0001), but not as an interaction term (Supplementary Figure S4). There were 12 bacteria that differed significantly by relative abundance in the overall model, seven best explained by age and 10 by ontogeny. *Frischella perrara* was significant in the model, but failed to meet significant thresholds after FDR correction. The bacteria *B. mellis*, *G. apis*, *Bifidobacterium indicum*, *B. asteroides*, *L. melliventris*, and *L. helsingborgensis* were explained by both factors in the model. *Bombilactobacillus mellis*, *B. asteroides,* and the two *Lactobacillus* were also very strongly correlated across all samples (Supplementary Table S5: Spearman’s *ρ*, *r_s_* > 0.60, *p* = 0.0004). These bacteria were negatively correlated with *G. apis* (Supplementary Table S5: Spearman’s *ρ*, *r_s_* < −0.19, *p* < 0.048) and *B. indicum* (Supplementary Table S5: Spearman’s *ρ*, *r_s_* < −0.44, *p* < 0.0004). *Gilliamella* sp. was explained by age in the MANOVA model and was most abundant in the 19 and 27-day-old bees (Supplementary Figure S7). Following patterns of typical or atypical ontogeny, an unclassified *Gilliamella*, an unclassified *Snodgrassella*, *L. kimbladii*, and the group of “SumOther” differed significantly. A Wilcoxon test of absolute abundance reveals the unclassified *Gilliamella* had greater abundance under conditions of typical ontogeny, while *L. kimbladii* and “SumOther” were greatest in atypical bees (Supplementary Figure S7). In contrast to the midgut, 16S/18S rRNA gene copy number in the ileum was relatively stable regardless of age or ontogeny (Supplementary Figures S4C,D). A PCA on CLR adjusted ileum microbiota shows more separation than the midgut for ontogeny, with 29.7% and 12.4% explained for the first and second component, respectively, (Figure 2C). Age-associated groupings are also consistent with the MANOVA where 27D bees break out from the other age groupings, especially in respect to *G. apis*, *Gilliamella* sp., and *B. indicum* (Supplementary Figure S5).

Correlations among the major gut species also reflects typical and atypical ontogeny. In the midgut, the abundance of *S. alvi* and *G. apicola* was positively correlated in workers with natural ontogeny (Supplementary Table S5), but showed no relationship in atypical PFs. *Gilliamella* sp. in PFs was also positively correlated with *F. fructosus* and *A. apinorum*, but in typical ontogeny these correlations were shared as well as strong positive correlations with *A. kunkeei*, *B. asteroides*, *L. apis*, *L. kimbladii*, and *S. alvi*. *Gilliamella apicola* and *Gilliamella* sp. absolute abundance did not correlate together significantly. In the ileum there were strong negative correlations between *G. apicola* and *G. apis* in all samples and groups (atypical vs. typical; Supplementary Table S6). An association with *S. alvi* and the unclassified *Gilliamella* group was found in typical ontogeny but missing in atypical PFs.

**Figure 3:**
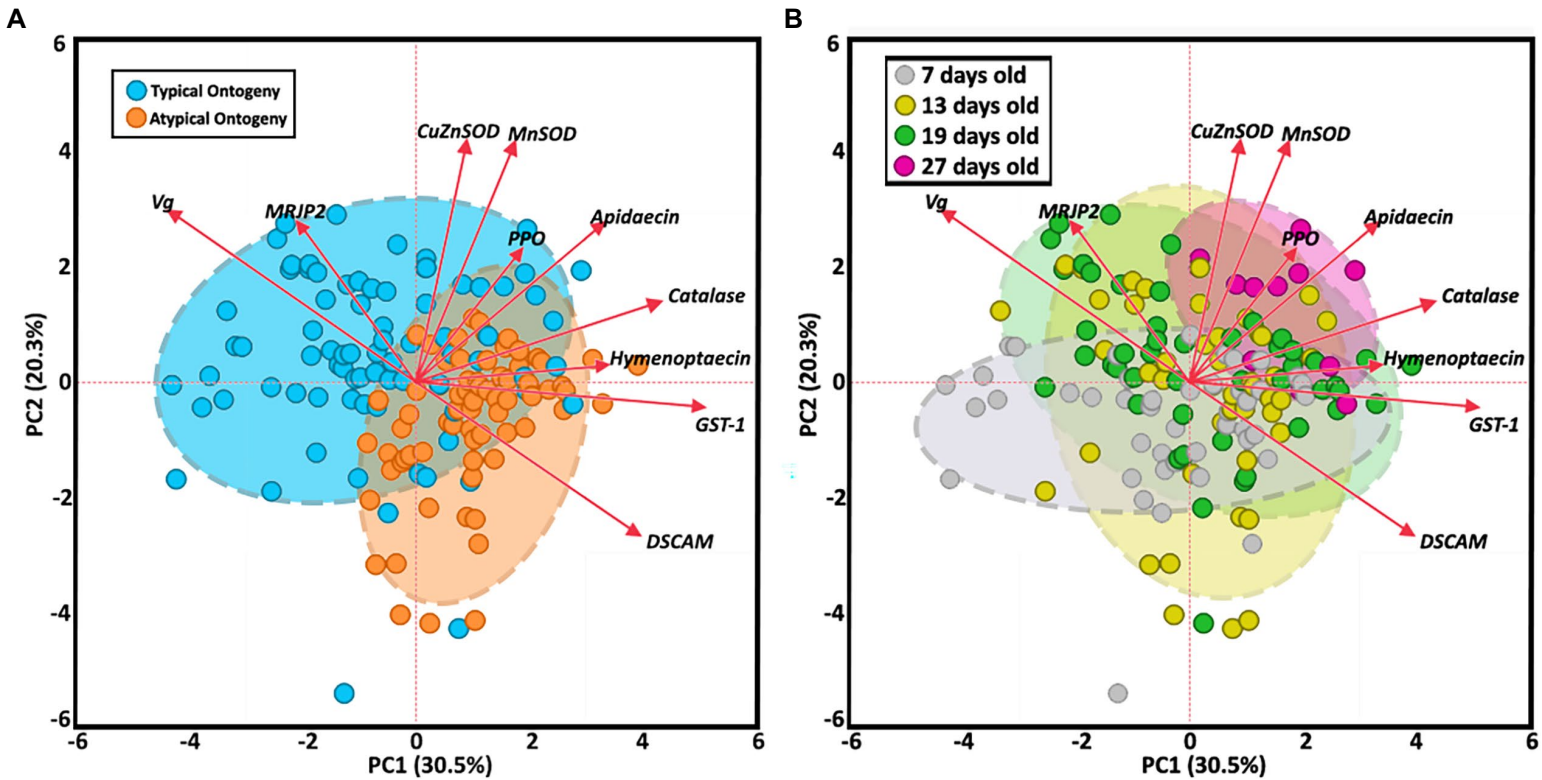
Principal component analysis of fat body gene expression by both ontogeny **(A)** and age **(B)**. Clustered groups of points contain similar gene expression. Longer vectors result from greater variation in gene expression. Density ellipses cover 90% of plots for each group.

**Figure 4:**
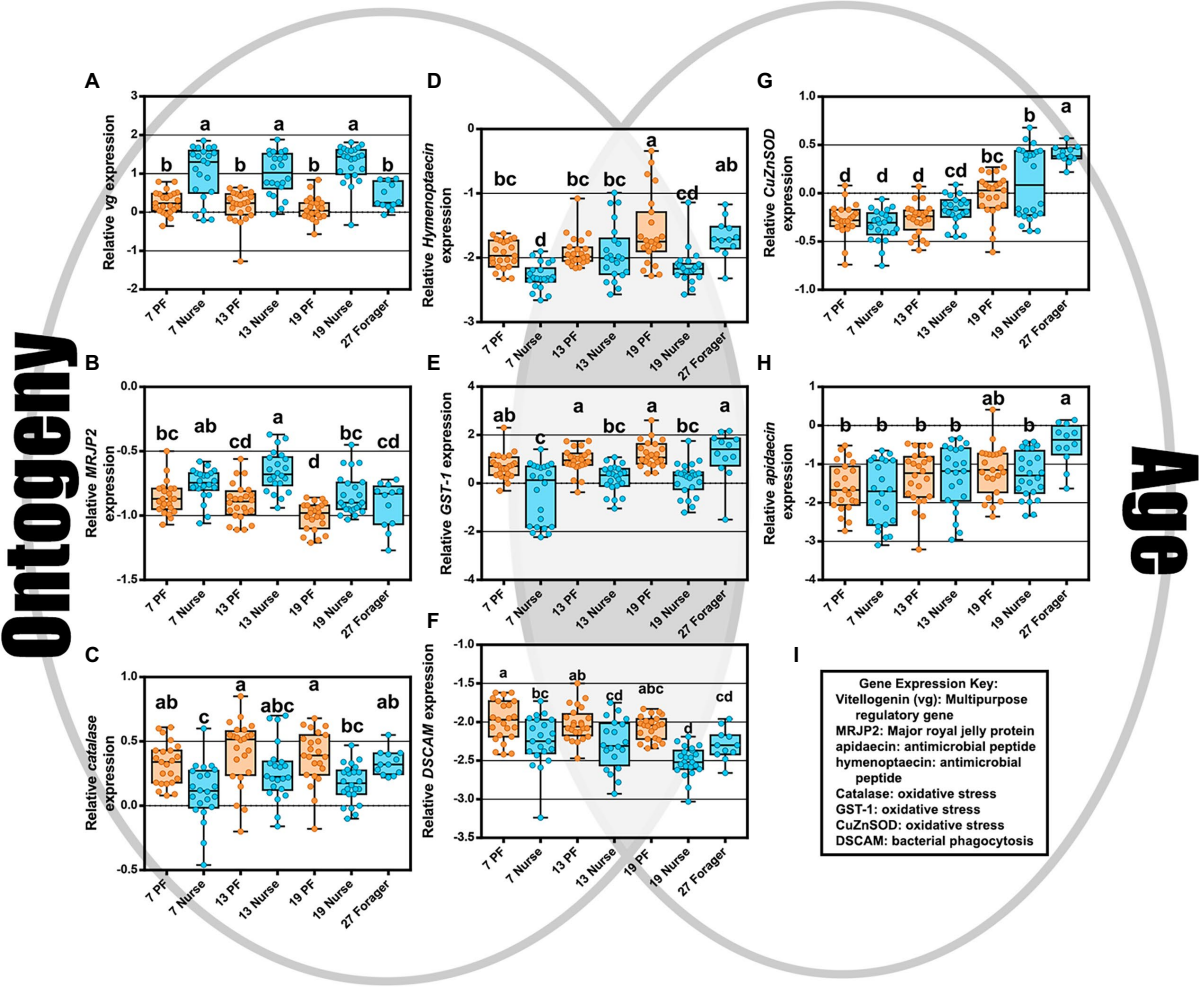
As depicted by the Venn diagram (gray background), gene expression reflects ontogeny, age, or an interaction of the two. **(A–C)** Vitellogenin, MRJP2, and catalase expression were associated with ontogeny. **(D–F)** hymenoptaecin, GST-1, and DSCAM expression were explained by either an interaction factor or combination of both ontogeny and age. **(G,H)** CuZnSOD and apidaecin expression increased with age regardless of ontogeny. **(I)** Key explaining functional roles of gene expression. Different letters indicate significant differences (*p* < 0.05).

The two-way MANOVA revealed a significant interaction effect of age and ontogeny for fat body gene expression (Supplementary Table S7). However, there were greater effects seen for individual gene differences by age or ontogeny. Two PCAs illustrate the relationship of immune gene expression with ontogeny and/or age (Figure 3). The first principal component (30.5%) maximizes the explained variance of immune gene expression, with gene vectors along the horizontal explaining greater variance associated with ontogeny (Figure 3A). In contrast, variation in gene expression distilled by the second component (20.3%) is more closely associated with age (Figure 3B). Overall, gene expression differed significantly by ontogeny or age or a combination of both factors (Figure 4). *Vitellogenin* and *MRJP2* are highly expressed in all age nurses and downregulated in age-matched and age-right foragers (Figures 4A,B). *Catalase*, *hymenoptaecin*, *GST-1*, and *DSCAM* had increased expression in atypical PFs relative to age-matched nurses following typical ontogeny (Figures 4C–F). Hymenoptaecin was significant by MANOVA for an interaction factor between ontogeny and age (Supplementary Figure S7), while *GST-1* and *DSCAM* were not. However, *GST-1* and *DSCAM* could be explained by either ontogeny or age independently and expression patterns show gene expression being controlled by both factors (Figures 4D–F). *CuZnSOD* and apidaecin followed age-associated gene expression patterns with age-right 27D foragers having the highest transcript levels (Figures 4G,H). *MnSOD* was also explained by an interaction factor in the MANOVA full model and *PPO* was the same across groups regardless of ontogeny or age (Supplementary Table S7).

We looked at Spearman’s *ρ* correlations between the midgut and ileum bacterial absolute abundances with fat body gene expression to investigate potential relationships between these tissues and overall immune health (Supplementary Tables S7–S10). In the midgut of atypical PFs, there was a negative correlation of *MRJP2* with *F. fructosus*, *A. kunkeei*, *Apilactobacillus apinorum*, and *Gilliamella* sp. For nurses (age 7, 13, and 19), there were significant correlations between *CuZnSOD* and *Gilliamella* sp., *A. kunkeei*, and *A. apinorum* and when considering typical ontogeny including age-right 27D foragers there were many additional positive relationships with *CuZnSOD*; *Gilliamella* sp., *A. kunkeei*, *A. apinorum*, *L. apis*, *L. melliventris*, *F. fructosus*, *S. alvi*, *B. mellis*, *B. asteroides*, and the collective sum of remaining bacteria “SumOther”. *Gilliamella* sp. was also negatively correlated with *MRJP2* and *Vg*, while positively correlated with *GST-1*, *apidaecin*, and *MnSOD*. *Hymenoptaecin* was also strongly correlated with *F. fructosus* and *A. kunkeei*.

In the ileum *Gilliamella* sp., *G. apis*, and the unclassified *Gilliamella* present significant correlations among bacteria and fat body gene expression (Supplementary Tables S9, S10). *Gilliamella* sp. was positively correlated with *CuZnSOD* in atypical PFs while *MnSOD* was positively correlated with *G. apis*. Again when considering typical ontogeny bees (including 27D) there were many more significant correlations. *CuZnSOD* was strongly correlated with *G. apis* and *Gilliamella* sp. *MnSOD* expression was strongly correlated with *G. apis*, *B. indicum*, and negatively correlated with *L. helsingborgensis*. Other significant negative correlations were *MRJP2* with *Gilliamella* sp. and *GST-1* with *L. helsingborgensis*. When considering all samples together there were more significant correlations, such as *Gilliamella* sp. being negatively correlated with *DSCAM* as well as the unclassified *Gilliamella*. The antimicrobial peptide *apidaecin* was also positively correlated with *Gilliamella* sp. and *B. indicum*. Interestingly, *B. asteroides* and *B. indicum* had a negative and positive correlation with *Vg* respectfully.

### Microbiota and gene associations with reversion

**Figure 5:**
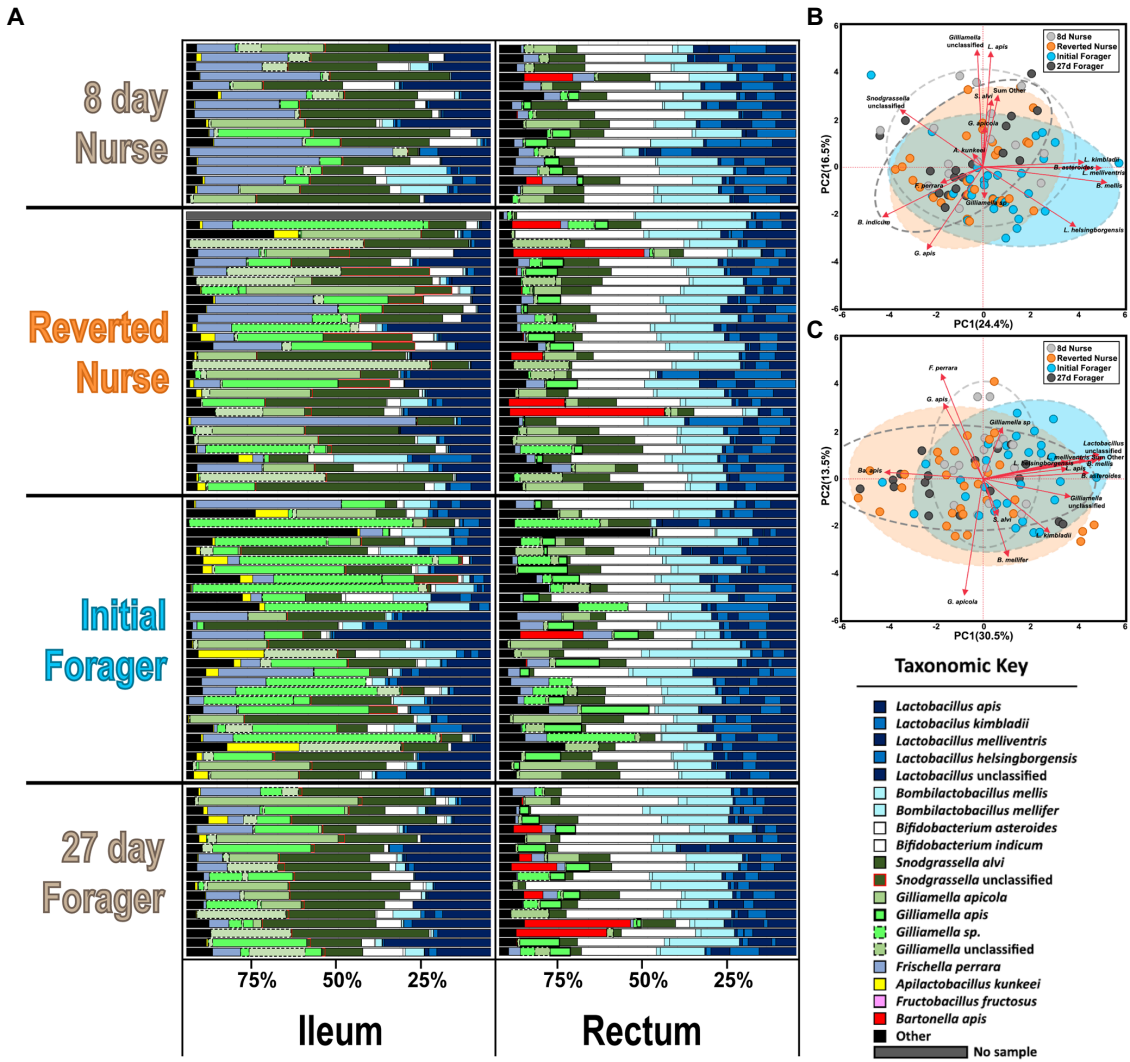
**(A)** Honey bee microbiota of the ileum and rectum. Results for the reversion experiment compare 8-day-old control nurses, 27-day-old control foragers, initial foragers (pre-reversion), and reverted nurses (initial foragers after 1 week of nursing). Color-coded bars represent relative abundance corrected by species-specific 16S rRNA gene copy number. Black represents diversity abundance (ileum: ASV’s 16–214, rectum: ASV’s 16–123). **(B,C)** Principal component analysis of the ileum and rectum, respectively, based on the most abundant ASVs. Clustered groups of points contain similar groupings of taxa with similar microbiota ratio abundances. Longer ASV vectors result from greater variation in CLR-adjusted scores. Density ellipses cover 90% of plots for each group.

A one-way MANOVA comparing the ileums and rectums of 8D and 27D old bees with the reversion experiment’s initial foragers (IF) and reverted nurses (REV) had some significant differences (Supplementary Figure S4). The overall MANOVA model in the ileum indicates significant differences by phenotype (*F* value 2.01, Pr > *F* = 0.0004) and for dependent variables: *S. alvi*, *F. perrara*, *L. kimbladii*, *L. melliventris*, and an unclassified *Snodgrassella*. Despite these significant values, the Wilcoxon results were not significant for any bacteria. The MANOVA model for the rectum was also significant (*F* value 2.1, Pr > *F* = 0.0002) with significant dependent variables *L. melliventris*, *L. apis*, *Bartonella apis*, *B. mellifer*, and an unclassified *Lactobacillus* group. The Wilcoxon found significant differences in the rectum when comparing all phenotypes together, but not when comparing reverted nurses to 8D nurses. Wilcoxon comparing REV with IF found a significant difference in the absolute abundance of *Ba. apis*, with REV having larger proportions. PCA on CLR adjusted ileum and rectum microbiota did not show as much separation in phenotypes as PF PCAs (Figures 5B,C). Groups shared significant overlap, but some differences seen in the rectum are explained solely by the presence of *Ba. apis* in several samples (Figure 5C). Bacterial load evaluated by total 16S copy number did not vary in the ileum or rectum (Supplementary Figure S4). Fungal load was also stable across phenotypes in the ileum, but were statistically significant in the rectum with 8D nurses and reverted nurses having the highest loads compared to 27D foragers and IF.

**Figure 6:**
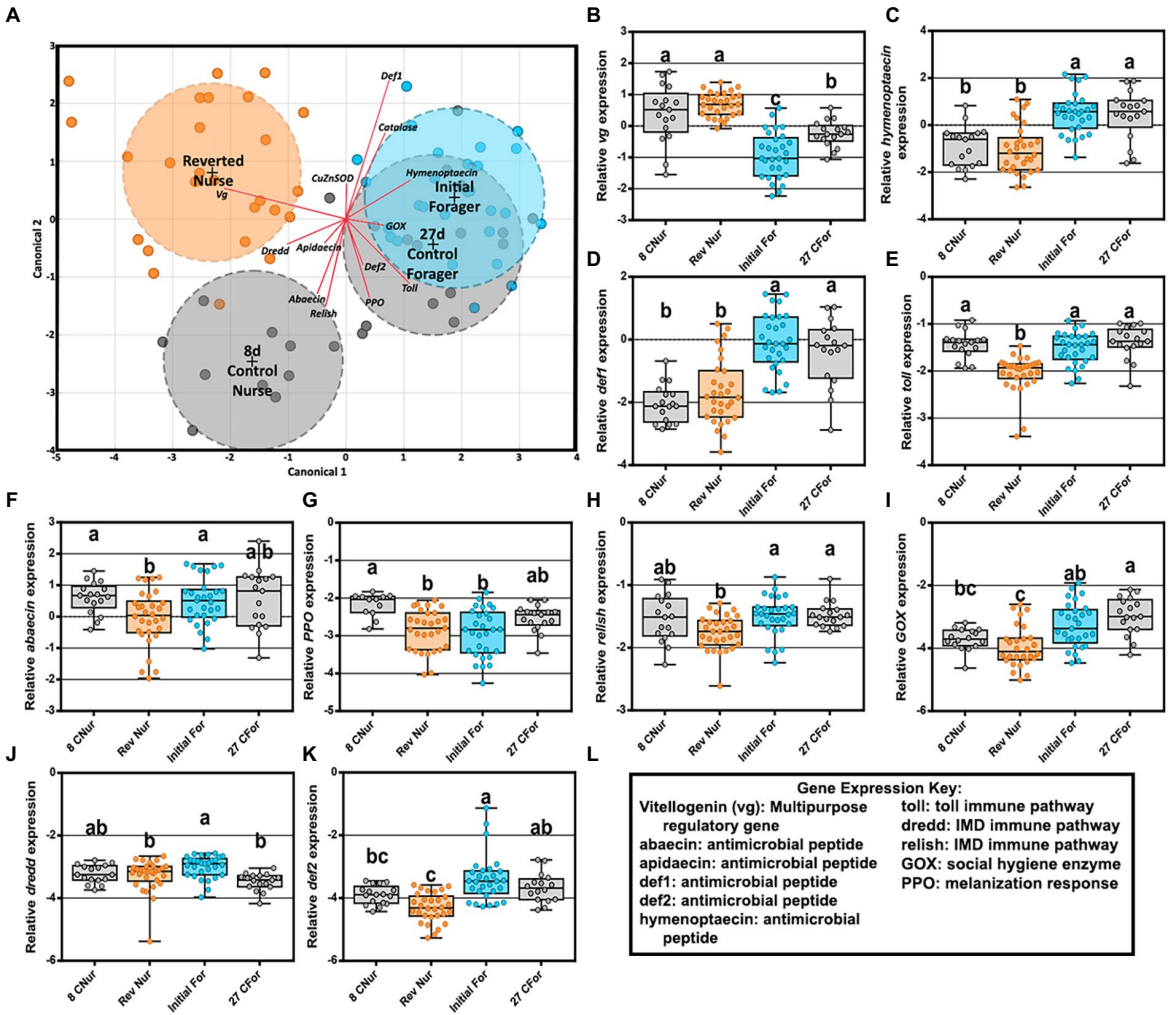
Reversion experiment gene expression comparing 8-day-old control nurses, 27-day-old control foragers, initial foragers (pre-reversion), and reverted nurses (initial foragers after 1 week of nursing) **(A)** Canonical correlation analysis of fat body gene expression by age and behavioral phenotype. Ellipses demarcate 95% confidence and phenotypes are displayed at the group’s centroid. **(B–D)** Fat body gene expression of reverted nurses was similar to that of 8-day-old control nurses. **(E–K)** Reverted nurse gene expression was reduced compared 8-day-old control nurses or foragers. **(L)** Key explaining functional roles of gene expression. Different lower case letters indicate significant differences (*p* < 0.05).

An LDA of fat body gene expression shows overlap of both forager phenotypes (27D and initial) but separation between 8D and REV (Figure 6A). Reverted nurse gene expression matched 8D nurses for increased *vitellogenin* expression and decreased for antimicrobial peptides *hymenoptaecin* and *defensin1* (Figures 6B–D). Other gene expression, including immune pathways or pathway–adjacent genes were either statistically lower relative to 8D, IF, and/or 27D bees or met a pattern of general depression (Figures 6E–L). Correlations between bacteria and gene expression in reverted nurses (Supplementary Tables S11, S12) had several positive interactions in the ileum. *Toll* expression positively correlated with *B. mellis*, *B. asteroides*, and *L. melliventris*, while *abaecin* expression was associated with *L. helsingborgensis* and *L. kimbladii* abundances. *Lactobacillus melliventris* was negatively correlated with *abaecin* in the rectum of REV, but generally across all phenotypes there were less correlations in the rectum.

### Carbonyl contents of abdomen

**Figure 7:**
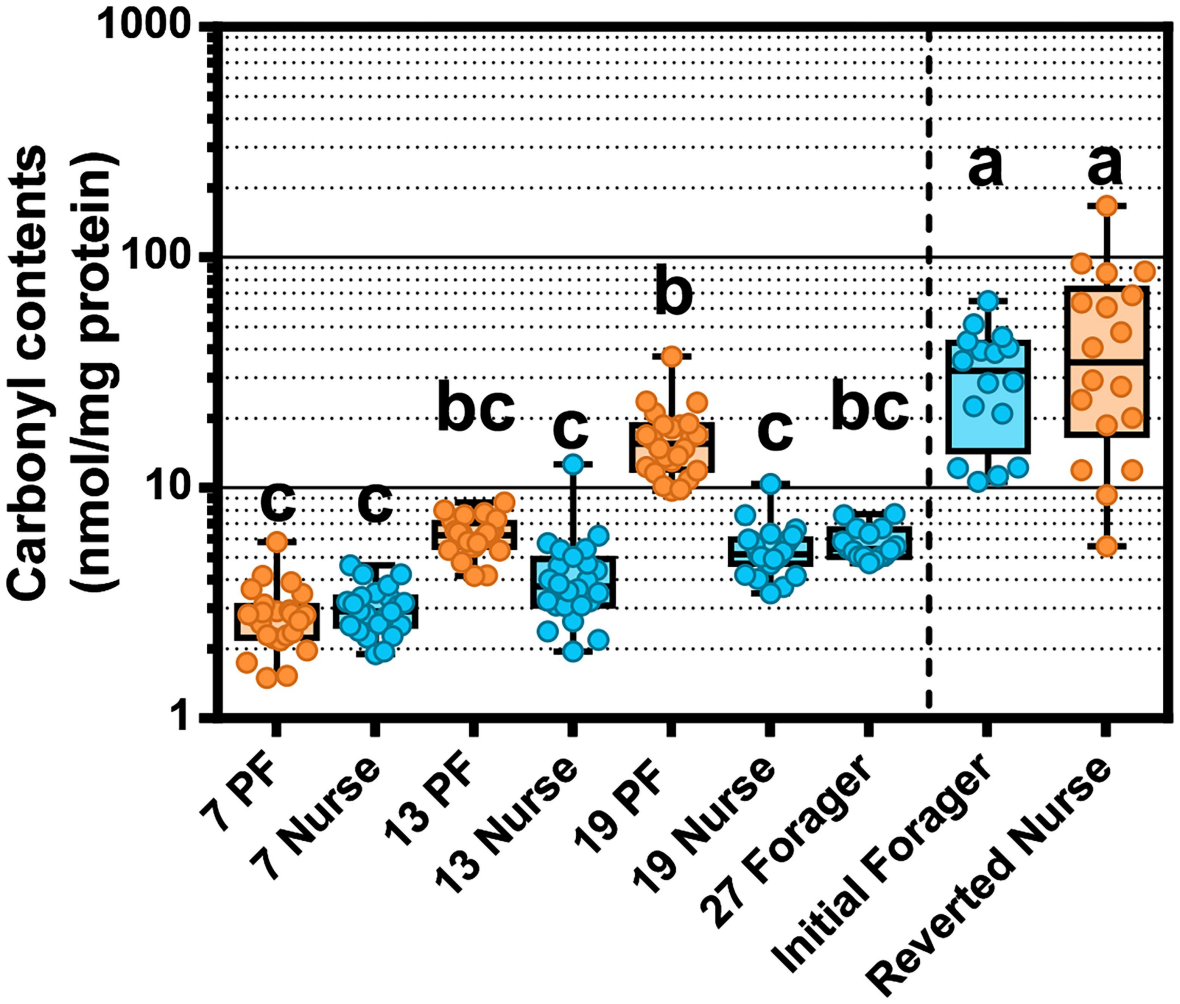
Oxidative stress measured as protein carbonylation in the worker fat body. Different letters indicate significant differences (*p* < 0.05). The line separates PF and REV experiments. REV initial foragers and reverted nurses have unknown ages, but were presumed to be the oldest bees across both experiments.

Oxidative damage in the abdomen was measured by carbonyl assay of the fat body and attached abdominal sclerites. PFs accumulated more oxidized proteins than age-matched nurses, a finding that became statistically meaningful after 19-days but started to trend in this direction at 13 days old (Figure 7). 19D PF had accrued significantly more carbonylation than 27D foragers following the typical ontogeny. Although we did not control for age in the REV study, IF and REV were biologically the oldest bees sampled. IF were presumed to follow typical ontogeny and 19D PF (known foragers since at least 7D) had reached similar levels of oxidative damage. As predicted Carbonyl contents decreased with increased Vg expression (*r_s_* = −0.30 *p* < 0.0005; Supplementary Table S12). To consider whether fat body gene expression or tissue bacterial absolute abundance had a relationship with carbonyl content accumulation we ran Spearman *ρ* correlation analyses (Supplementary Tables S7–S10). In the midgut, carbonyl contents were positively correlated with *S. alvi* in atypical PFs but also *Gilliamella* sp., *L. apis*, *F. fructosus*, *F. perrara*, *B. mellis*, and the “SumOther” across all samples. In the ileum, atypical PFs had carbonyl positively correlated with *Gilliamella* sp. and *L. apis.* The strong correlation with *Gilliamella* sp. also carried over to correlations across all samples, but interestingly there were also many correlations with carbonyl with typical ontogeny. Carbonyl had strong positive correlations with *Gilliamella* sp., *L. apis*, *G. apis*, and “SumOther” and a strong negative correlation with *L. helsingborgensis*.

## Discussion

Division of labor and phenotypic plasticity are the hallmarks of social insect success (Hölldobler and Wilson, 1990; Fewell, 2003). In the experiments detailed here, we manipulated colony-level task allocation to investigate effects on tissue-specific microbiome succession and associated physiology (Figure 1). During typical adult ontogeny, worker honey bees undergo an age-based division of labor, transitioning from in-hive tasks to riskier foraging behavior as they age. The natural acquisition, and succession, of the gut microbiome has been described throughout this process, a data set biased toward young nurse-aged bees (Sabree et al., 2012; Corby-Harris et al., 2014; Kwong and Moran, 2016; Anderson et al., 2018; Copeland et al., 2022). Our results provide a new perspective on the aging microbiota, detailing tissue-specific results from a large sample of workers aged >19 days. In the reversion (REV) experiment, we induced a social reversion of foragers back to a nursing behavioral phenotype, requiring a second round of pollen consumption by older aging workers. The precocious forager (PF) experiment induced workers to forage much earlier than normal, highlighting a common colony-level phenotype associated with various forms of colony dwindling (Perry et al., 2015).

Early nutritional state is associated with typical adult ontogeny in many social insects including honey bees (Amdam et al., 2004; Anderson et al., 2008). In early adult workers, the somewhat constitutive expression of vitellogenin (Vg) following pollen consumption is associated with insulin-like peptide signaling and the presence of the hindgut microbiome (Zheng et al., 2017). In turn, the Vg rich environment found in the hive provides social immune function and extended resistance to oxidative stress in the hemolymph (Amdam et al., 2003; Seehuus et al., 2006; Corona et al., 2007). A worker that does not engage in foraging can live for many months to nearly a year (Sakagami and Fukuda, 1968). This extended period within the hive also allows partner choice among core gut bacterial species of *Gilliamella* for *Snodgrassella* providing favorable microbiome function (Kwong et al., 2017; Zheng et al., 2017). Unlike the other core microbiota members, *Gilliamella* establishment is strongly influenced by diet and social interaction, perhaps even social immunity (Powell et al., 2014; Anderson et al., 2016, 2022). The factors that contribute to increased *Gilliamella* dominance in the aging ileum require more investigation. Past work had assumed complete hindgut assembly by 7–9 days of age, a time point defined by a high frequency of nurse duties, but our results here (Figure 2) and in the literature (Moran et al., 2012; Horton et al., 2015) indicate that *Gilliamella* establishment in the ileum can require >13 days.

Our experimental design was successful in quantifying the typical physiology and microbiota of adult worker development capturing the natural transition from nurse to forager. Associated with the control of complex social behaviors in honey bees, Vg is tightly linked to division of labor, patterns of oxidative stress and immune gene expression, and succession of the gut microbiota. As revealed by the carbonyl assay, the accumulation of oxidative stress was a function of Vg expression. As the primary exemplar, mated queens can withstand extreme oxidative stress without loss of function. Mated queen phenotypes are constantly fed royal jelly, and thus high Vg titers circulate constantly throughout their hemolymph providing protection from oxidative damage (Amdam et al., 2003; Seehuus et al., 2006; Corona et al., 2007). Here we found a similar function for Vg in reverted nurses, wherein investment in oxidative stress and immunity was significantly depleted in the high Vg environment (Figure 6B).

A number of significant relationships between microbial species abundance and fatbody gene expression support our post-hoc hypothesis; that oxidative stress or immune gene expression predicts microbial taxonomy and abundance. Beyond relationships demonstrated previously (Zheng et al., 2016, 2017; Emery et al., 2017; Kwong et al., 2017), we note a relationship of *Gilliamella apis* abundance in the ileum with the expression of CuZnSOD, and carbonyl accumulation in precocious foragers (Table 1). Giving context to this relationship, *Gilliamella apis* is also associated with old age, becoming the dominant *Gilliamella* in the ileums of workers aged 27 days and older (Figure 5). In contrast, early ileum colonization was dominated by *G. apicola*. While *G. apicola* maintains its numbers with age, both *G. apis* and an unclassified *Gilliamella* species begin to reproduce in older workers beginning at 13 and 19 days, respectively (Figure 2). Competition between *G. apicola* and *G. apis* is supported by significant negative correlations within individual ileums, but in general, all four *Gilliamella* species either remain stable or increase in absolute abundance with age. We suggest that larger and more diverse *Gilliamella* populations are supported by changes in host physiology and/or diet that accompany aging.

**Table 1:** Summary of Spearman rank correlations of ileum OTU absolute abundance, carbonyl, and fat body gene expression that were determined significant after FDR correction (*p* < 0.05) for various phenotypes.

Phenotypes included in spearman *ρ* correlations	Gene expression or carbonyl	Bacterial species	Spearman *ρ*	Prob > |*ρ*|	Plot
All samples 7PF, 7Nu, 13PF, 13Nu, 19PF, 19Nu, 27For	Carbonyl	*Lactobacillus apis*	0.4457	0.0008	++++
	GST-1	*Lactobacillus apis*	0.4295	0.0008	++++
	CuZnSOD	*Gilliamella* sp.	0.4105	0.0008	++++
	CuZnSOD	*Gilliamella apis*	0.3913	0.0008	++++
	Vitellogenin	*Lactobacillus apis*	−0.3497	0.0015	−−−
	Carbonyl	SumOther	0.3335	0.0021	+++
	Carbonyl	*Gilliamella* sp.	0.3122	0.0048	+++
	MNSOD	*Gilliamella apis*	0.3167	0.0048	+++
	Apidaecin	*Lactobacillus apis*	0.3093	0.0062	+++
	MRJP2	*Bifidobacterium asteroides*	−0.3057	0.0069	−−−
	MRJP2	*Lactobacillus apis*	−0.3036	0.0073	−−−
Atypical ontogeny 7PF, 13PF, 19PF	Carbonyl	*Gilliamella* sp.	0.6307	0.0035	++++++
	CuZnSOD	*Gilliamella* sp.	0.4437	0.0281	++++
	Carbonyl	*Lactobacillus apis*	0.4347	0.0305	++++
	MRJP2	*Apilactobacillus kunkeei*	−0.4525	0.0222	−−−−−
Typical ontogeny 7Nu, 13Nu, 19Nu	Vitellogenin	*Lactobacillus apis*	−0.5714	0.0023	−−−−−−
	GST-1	*Lactobacillus apis*	0.4911	0.0067	+++++
	MnSOD	*Gilliamella apis*	0.4628	0.0113	+++++
	Carbonyl	*Lactobacillus helsingborgensis*	−0.4532	0.014	−−−−
Typical ontogeny 7Nu, 13Nu, 19Nu, 27For	Vitellogenin	*Lactobacillus apis*	−0.5595	0.0013	−−−−−−
	MnSOD	*Gilliamella apis*	0.4779	0.0013	+++++
	CuZnSOD	*Gilliamella apis*	0.4562	0.0025	+++++
	GST-1	*Lactobacillus apis*	0.4398	0.0041	++++
	Carbonyl	*Lactobacillus helsingborgensis*	−0.4218	0.005	−−−−
	Carbonyl	*Gilliamella* sp.	0.4097	0.0074	++++
	Carbonyl	*Lactobacillus apis*	0.4034	0.0088	++++
	MnSOD	*Bifidobacterium indicum*	0.3796	0.0193	++++
	CuZnSOD	*Gilliamella* sp.	0.3628	0.0281	++++
	Carbonyl	SumOther	0.3449	0.031	+++
	MRJP2	*Gilliamella sp.*	−0.3519	0.0328	−−−−
	GST-1	*Lactobacillus helsingborgensis*	−0.3439	0.0378	−−−
	CuZnSOD	*Snodgrassella* unclassified	0.3342	0.0444	+++
	Carbonyl	*Gilliamella apis*	0.3211	0.046	+++

### Reversion experiment

Given the established relationship between vitellogenin expression and the hindgut microbiome (Zheng et al., 2017), we predicted that old foragers reverting to a nursing phenotype would harbor a healthy nurse-like hindgut microbiome. During this transition, we found that the ileum and rectum (hindgut) microbiotas remained remarkably stable (Figure 5) suggesting that the established biofilm had attained a climax community resistant to host physiological changes. Given their high nutritional state, the reverted nurses may not require the expression of oxidative stress genes or immune genes at levels matching a young nurse. Foragers subsist primarily on simple sugars; nectar and honey. For a forager to revert to a nurse, a second round of pollen consumption is required to meet the physiological demands of nursing. Gene expression profiles were distinct between reverted nurse, young nurse and older forager phenotypes (Figure 6A) reflecting differences in the expression of antimicrobial peptides (AMPs), oxidative stress, and innate immunity genes. Both forager groups shared significant gene expression by task at 27 days. Although reverted nurses were the oldest bees in the study, they had the greatest Vg expression (Figure 6B), even higher than 8 day old nurses. This study and others show that foragers typically have increased expression of AMPs and oxidative stress genes relative to nurses (Vannette et al., 2015; Cervoni et al., 2017). In contrast, with the exception of Vg, gene expression from reverted nurses was mostly depleted or depressed in relation to both younger nurses and foragers (Figures 6C–K).

The expression differences between young and old nurses are best explained with reference to vitellogenin expression and carbonyl content. The carbonylation in worker fat body reflects the oxidation of Vg as compared to other storage proteins, such that a direct relationship is evident in our results (Figure 7). Reverted nurses showed the greatest Vg expression and the greatest ratio of oxidized carbon atoms relative to general protein. Similar to mated queen phenotypes, Vg provides major long-term oxidative stress management for the insect (Fedorova et al., 2014; Salmela et al., 2016).

### Precocious foragers experiment

At the other phenotypic extreme, precocious foragers (PFs) experience a rapid depletion of Vg and were less likely to possess *Gilliamella* spp. partnered with *Snodgrassella* spp. in the ileum contributing to gut dysbiosis. Based on results obtained with the genes selected for this analysis, host physiology was best explained by an interaction of age and ontogeny, with the greatest variation explained by ontogeny (Supplementary Table S7). This result is likely a reflection of differential nutrition and Vg titers associated with both age and task performance (Figure 4). We found that Vg was already differentially expressed by day 7 for same-age nurses and PFs (Figure 4A). Thus, Vg expression and task specialization influenced the trajectory of microbiome succession and fat body gene expression in typical versus atypical ontogeny paths. PFs with low Vg expression and poorly developed ileum microbiomes incur oxidative damage *via* the accumulation of carbonyl contents in the hemolymph at a significantly greater rate than both age-matched nurses and age-right (27D) foragers (Figure 7). Young bees transition to foraging faster when there is limited social contact with older bees (Huang and Robinson, 1996; Pankiw, 2004). This can occur in response to various biotic (predators, pathogens) or abiotic pressures including pesticides and anthropogenic factors. The nutrient deficient physiology of PFs may be poorly suited for tasks outside the hive (Vance et al., 2009) and evidence suggest that PF individual risk of death increases relative to older foragers (Prado et al., 2020). Precocious foraging is also less productive (Chang et al., 2015), factors that conspire to accelerate colony loss (Perry et al., 2015).

Antimicrobial peptides (AMPs) are expressed as part of the innate immune system of the honey bee (Alberoni et al., 2016). Explained primarily by ontogeny, we found high levels of hymenoptaecin in all ages of foragers and strong upregulation of apidaecin in 27D foragers. This pattern is supported by studies that show foragers express genes encoding AMPs in greater abundance than nurses (Vannette et al., 2015). Similar to our findings, honey bees inoculated with gut microbiota or mono-colonized with *S. alvi*, upregulated apidaecin and hymenoptaecin constitutively in the fat body (Kwong et al., 2017). The core microbiota tends to have increased tolerance for host AMPs compared to non-native microorganisms (Kwong et al., 2017), therefore it’s advantageous to constitutively express AMPs as a prophylactic measure given that foragers are exposed to more pathogen pressure outside the hive. Younger foragers also expressed DSCAM higher than age-matched nurses, with an age-associated decline by 19D (Figure 4F). In *Drosophila*, DSCAM is a highly diverse Ig-superfamily receptor that may affect phagocytic uptake of bacteria by host hemocytes (Watson et al., 2005). The number of honey bee hemocytes decrease in relation to age and behavioral task (Amdam et al., 2004, 2005; Schmid et al., 2008), thus, foragers have decreased hemocyte counts in the hemolymph and a higher number of pycnotic cells than nurse bees. The honey bee DSCAM gene has the potential to generate as many as 12,000 splice variants which may allow them to target specific microorganisms (Graveley et al., 2004). The expression of DSCAM shows a strong negative association with Vg expression (Supplementary Table S13), suggesting it is not governed by nutritional state. Taken together, higher DSCAM expression in nutrient depleted foragers may serve to increase efficiency of the decreased number of hemocytes.

We also considered the effects of oxidative stress in relation to aging and ontogeny. Oxidative stress, produced by intensive foraging flights, is likely mitigated by host enzyme expression within the limits of host physiology. We found that CuZnSOD increased with age, and catalase and GST-1 were highest in precocious foragers. CuZnSOD detoxifies the free radical superoxide (O_2_·-) into the less reactive hydrogen peroxide (H_2_O_2_), which is then processed by catalase into water and oxygen (Lei et al., 2016). Mitochondrial activity during aerobic respiration (flight) is the main cause of ROS generation, which tends to generate more H_2_O_2_. Fat body respiration is greater in nurses due to the continuous metabolic function needed to sustain brood rearing. Foragers experience ROS production in the flight muscles of the thorax that would circulate throughout the hemolymph. A previous study found that abdominal H_2_O_2_ levels were elevated in forager flight muscles as a likely result of increased mitochondria density (Cervoni et al., 2017). Foragers also have decreased abdominal lipid stores (Toth and Robinson, 2005), less developed fat body (Ament et al., 2008; Wilson-Rich et al., 2008), and a decrease in Vg expression compared to nurses (Seehuus et al., 2006). While young bees have generally more resistance to oxidative stress, foragers incur a gradual accumulation of tissue damage reflecting age-associated declines in the efficiency and degradation of ROS (Williams et al., 2008). We showed that 19D PF that had been foraging since at least 6-days old, had a higher level of fat body protein carbonylation relative to age-matched nurses and 27D foragers (Figure 7). The age-right 27-day foragers had the highest oxidative stress gene expression, which could explain their low levels of protein carbonylation. These were the bees that remained nurses the longest and most recently transitioned to foraging (see Vg expression relative to 7–13–19-day foragers Figure 4A). The 19D PFs antioxidant capacity may have reached its physiological limit. Paradoxically, ROS can have positive effects such as acting in redox signaling pathways (Lei et al., 2016) or modulating the microbiota (Engel and Moran, 2013b). Our results suggest that the physiological cost of early foraging is extreme, and highlight the progression of colony dwindling, a common but poorly understood process.

The midgut microbiota varied in composition based on age and ontogeny (Figure 2; Supplementary Table S4). In agreement with past results (Anderson and Maes, 2022) we report the aging midgut as a potential niche for microbial invasion (Figures 3, 4). It has been suggested that the midgut is inhospitable to microbial colonization due to the continual shedding of the peritrophic membrane, however, recently it was shown that the peritrophic membrane is absent or greatly reduced in foragers (Harwood and Amdam, 2021), which may leave the tissue vulnerable to microbial opportunism. We saw a massive increase in midgut microbiome size in 27D foragers, an order of magnitude larger than younger bees including significantly more (non-core) bacterial diversity (Supplementary Figure S4); a trend observed as early as 7D (Supplementary Figure S4). *Gilliamella apicola* and *Gilliamella* sp. absolute abundance in the midgut was greatest in age-matched nurses vs. foragers and comprised nearly half of bacterial cells in 27D foragers. Likewise, CuZnSOD expression levels were associated with both bacterial cell abundance and *Gilliamella* spp. in the midgut (Figure 4; Supplementary Tables S7, S8). *Gilliamella* often dominates the midgut (Ludvigsen et al., 2015), but can be lacking in the ileums of young nurse workers, becoming better established at middle age (Anderson and Ricigliano, 2017). Strains of *Gilliamella* have varying capabilities to degrade pollen cell wall components (Engel and Moran, 2013a), metabolize toxic monosaccharides (Zheng et al., 2016), and encode partner compatibility genes such as type VI secretion systems (Steele et al., 2017). *Gilliamella apis* and the unclassified *Gilliamella* abundances in the ileum showed the strongest relationship with ontogeny, establishing more efficiently in nurses than precocious foragers. As hypothesized previously (Anderson and Maes, 2022) performing tasks within the hive improves the chance of compatible *Gilliamella* establishment, or fortifies its establishment *via* other mechanisms.

Midgut bacterial growth of 27 day old foragers was characterized by increased diversity abundance coupled with blooms of *A. kunkeei* and *F. fructosus* and an unclassified species of *Gilliamella*. Coculture assays demonstrate that *A. kunkeei* and *F. fructosus* support the growth of other honey bee symbionts considered “core hindgut bacteria” (Rokop et al., 2015). Together, this suggests that some so-called transient microbes likely have co-evolved functional roles within the honey bee gut as more ubiquitous microbial members. In the midgut, samples often had a dynamic mix of *Lactobacillus* comprised of at least 3–4 strains in varying proportions. However, the ileum was largely dominated by *Lactobacillus apis* which should be considered the *de facto* ileal strain. This is supported by previous research indicating there are a variety of host adapted strains of *Lactobacillus* Firm5 in the system, some more proximately available to colonize niches during age-based succession (Anderson et al., 2016). Additionally, *L. apis* is a pioneer species able to populate the honey bee ileum and maintain dominance even as its relative abundance gives way to other core members. *Lactobacillus apis* was also associated with carbonyl accumulation and negatively associated with Vg expression suggesting a direct relationship with the aging ileum and overall host senescence.

The ileal *Gilliamella/Snodgrassella* relationship is one primary metric of a healthy gut microbiome (Zheng et al., 2017). For the adult worker, *S. alvi* is considered a keystone species in the ileum/pylorus, interfacing with host epithelium and creating a protective biofilm with *G. apicola* and *Lactobacillus apis* (Martinson et al., 2012). *Snodgrassella alvi* protects the host from opportunism (Maes et al., 2016), while consuming oxygen and producing nutrients to support other gut bacteria (Kwong and Moran, 2016). Both *G. apicola* and *S. alvi* increase with age and stabilize in ratio abundance in the midgut and ileum, succession apparently accelerated and reinforced by an extended nursing role in early life (Supplementary Tables S6, S7). The succession of gut bacteria in honey bee workers is typically considered a climax community at 7–9 days of adult life. However, we found that variation of the three major ileum bacteria was minimized, and evenness of the entire microbiome maximized at 27 days of age, suggesting the attainment of a climax community that provides the greatest protection for new foragers. The different successional patterns documented between midgut and ileum might suggest an important function of the gut microbiome in mitigating gut opportunism and dysbiosis. Following the decreased production of midgut peritrophic membrane, bacteria populate the midgut, a process that may rely on early bacterial succession and strong establishment of core ileum species, as seen with normal ontogeny. We speculate that the co-evolved character of ileum bacteria present at this transition may contribute to longevity or accelerate opportunistic diseases like *Nosema*. Natural or premature immunosenescence provides context for how immunity shapes and is shaped by the host microbiota. Host regulation/dysregulation of the microbiome resulting in the reduction of *Lactobacillus* spp. and expansion of *Gilliamella* spp. with age requires more investigation.

## Conclusion

Here we found that the social structure of honeybee colonies affects composition of the gut microbiota and associated metabolism. Honey bees are challenged continuously by environmental and agricultural factors that alter colony demography. Occurring with *Nosema* disease, pollen dearth, pesticide exposure and viral disease, precocious foraging is a widespread colony-level deficiency. Worker aging in honey bees can be defined as the ratio between vitellogenin levels and oxidative stress. High vitellogenin titers in reverted nurse phenotypes compensated for decreased gene expression associated with immunity and oxidative stress. Premature foragers quickly accrue oxidative damage as a result of intense foraging activity and low Vg levels. The physiological demands of foraging are best met by older workers that have transitioned to foraging following extended development within the hive. The ratio abundance of keystone ileum species *S. alvi* and *G. apicola* is refined with an extended nursing period within the hive, setting the stage for long-lived foragers. Our study highlights the importance of tissue-specific microbiome sampling, revealing niche specialization of bacterial species. In agreement with previous hypotheses (Anderson and Ricigliano, 2017), our results indicate that the aging midgut becomes a niche for rapid microbial colonization, with potential consequences for both individual and colony survival.

## Data availability statement

The data presented in the study are deposited in GenBank, Sequence Read Archive, accession numbers PRJNA801240 and PRJNA885470.

## Author contributions

DC, PM, BM, and KA contributed to experimental design and commented on the manuscript. DC and PM performed laboratory work with the input of KA and BM. DC and KA analyzed the data and wrote the manuscript. All authors contributed to the article and approved the submitted version.

## Funding

The study was supported by the ARS-USDA, Carl Hayden Bee Research Center, CRIS project plan Anderson 2022-21000-021-00D. The ARS is an equal opportunity employer and provider.

## Conflict of interest

The authors declare that the research was conducted in the absence of any commercial or financial relationships that could be construed as a potential conflict of interest.

## Publisher’s note

All claims expressed in this article are solely those of the authors and do not necessarily represent those of their affiliated organizations, or those of the publisher, the editors and the reviewers. Any product that may be evaluated in this article, or claim that may be made by its manufacturer, is not guaranteed or endorsed by the publisher.

## Supplementary material

The Supplementary material for this article can be found online at: https://www.frontiersin.org/articles/10.3389/fmicb.2022.1059001/full#supplementary-material
